# Mesenchymal stromal cells isolated from chicken peripheral blood secrete bioactive factors with antimicrobial and regenerative properties

**DOI:** 10.3389/fvets.2022.949836

**Published:** 2022-08-24

**Authors:** Rebecca M. Harman, Katherine A. Churchill, Sonia Parmar, Gerlinde R. Van de Walle

**Affiliations:** Baker Institute for Animal Health, College of Veterinary Medicine, Cornell University, Ithaca, NY, United States

**Keywords:** chicken, mesenchymal stromal cells, peripheral blood, secreted factors, regenerative, antimicrobial

## Abstract

Mesenchymal stromal cells (MSCs) are adult multipotent progenitor cells that have been isolated from various tissue sources of many species, primarily mammals. Generally, these cells proliferate extensively in culture and have been shown to secrete bioactive factors that contribute to healing processes by regulating inflammation, modulating immune responses, inhibiting bacterial growth, and promoting tissue regeneration. The present study reports on the isolation and characterization of MSCs from the peripheral blood (PB) of chickens. Chicken PBMSCs were characterized based on their trilineage differentiation potential and gene and protein expression of MSC-specific cell surface markers. To determine functionality, conditioned medium (CM), which contains all bioactive factors secreted by MSCs, was collected from chicken PBMSCs, and used in *in vitro* antimicrobial, migration, and angiogenesis assays. Chicken PBMSC CM was found to (i) inhibit the growth of planktonic *Staphylococcus aureus* (*S. aureus*), and even more significantly the methicillin-resistant *S. aureus* (*MRSA*), (ii) decrease adhesion and promote migration of fibroblasts, and (iii) support endothelial cell tube formation. Collectively, these data indicate that chicken PBMSCs secrete bioactive factors with antimicrobial and regenerative properties, and as such, provide a novel source of cell-based therapies for the poultry industry.

## Introduction

Mesenchymal stromal cells (MSCs) are adult, multipotent progenitor cells that have been isolated from many species, including chickens (1). In chickens, MSCs have previously been collected from bone marrow, umbilical cord, skin, amniotic tissue, lung and intestine (2–12). These chicken MSCs exhibited the potential to differentiate into adipocytes, chondrocytes, and osteocytes, and have been reported to express the cell surface markers CD29, CD44, CD73 and CD90, but not CD31 and CD34, consistent with MSC data from other species. Moreover, chicken MSCs have been shown to have the potential to differentiate into beta-like pancreatic islet and spermatogonial cells (6, 13). Functionally, chicken bone marrow (BM)-derived MSC were found to exhibit an anti-tumoral effect by inducing apoptosis in tumor cell lines (3), and chicken lung-derived MSC suppressed T-cell activity (11).

Our group has previously shown that equine peripheral blood-derived MSCs (PBMSCs) act on target cells *in vitro* in ways that suggest they may promote wound healing *in vivo*, for example healing of chronic, non-healing wounds. Specifically, we demonstrated that conditioned medium (CM) collected from equine PBMSCs, which contains all factors secreted by these cells and is named the MSC secretome, can inhibit bacterial growth, decrease adhesion and increase migration of equine fibroblasts, and promote angiogenesis (14–18).

Based on our previous findings with the equine PBMSC secretome, the current study was designed to explore the potential of chicken PBMSC CM as a novel biological for the treatment of cutaneous diseases of poultry. Both commercially-raised and backyard chickens suffer from a variety of infectious skin conditions, such as gangrenous dermatitis (GD) and pododermatitis or bumblefoot. GD is an economically important disease of domestic chickens and turkeys, caused by anaerobic or aerobic bacteria (19). Clinical cutaneous signs of GD include featherless skin, subcutaneous edema, ulcers, and necrotic skin tissue (20, 21). GD is traditionally treated by adding antibiotics to drinking water or feed (22), but over the counter topical antibiotic sprays are also available to reduce the bacterial load in the skin of chickens. Pododermatitis is a degenerative, inflammatory condition of the bottom of the foot in birds, characterized by ulcerations, that can advance to deeper infections, affecting tendons and digital bones (23, 24). Contributing factors include inactivity, housing that encourages abnormal weight-bearing, poor nutrition, and poor husbandry (24, 25), and both prevention and treatment of pododermatitis consist of a multimodal approach aimed at correcting the underlying problems (24). Chicken PBMSC CM therapy could contribute to either of these conditions, further improving the quality of life of poultry and save industry resources.

## Materials and methods

### Cell isolation and culture

Blood collection was approved by the Cornell Institutional Animal Care and Use Committee (IACUC # 2014-0038). Chicken peripheral blood (PB) mononuclear cells (PBMCs) and mesenchymal stromal cells (PBMSCs) were isolated from PB of laying hens using Ficoll (GE Healthcare, Chicago, IL), as described previously for equine PBMCs and PBMSCs (26, 27). Chicken PBMSCs (*n* = 3) were cultured in medium consisting of Dulbecco's Modified Eagle Medium (DMEM) (Corning Life Sciences, Lowell, MA), supplemented with 30% fetal bovine serum (FBS) (Bi-Techne, Minneapolis, MN), 2% chicken plasma, 2 × 10^−12^ M dexamethasone (Sigma Aldrich, St. Louis, MO), and 1x penicillin-streptomycin (P/S) (Corning Life Sciences). After the first passage, cells were cultured in similar medium but without dexamethasone (Chicken MSC Culture Medium).

Chicken aortic endothelial cells (AECs) were isolated from 18-day-old chicken eggs, using a protocol developed by Lion et al. (28). Briefly, aortic vessels were dissected from fetal hearts, minced, and plated in petri dishes coated with 0.2% gelatin type B (Sigma Aldrich) in EGM-2MV medium (Lonza, Shady Grove, MD). After 2 days of incubation, aortic vessels were gently removed from wells using phosphate buffered saline (PBS), and fresh EGM-2MV medium was added to the dishes. Cells that had migrated out of the vessels were maintained as an adherent culture, changing the medium every other day and passaging after 3–4 days. After the first passage, AECs were further cultured on non-gelatin coated culture flasks.

The chicken fetal fibroblast cell line UMNSAH/DF-1 (ATCC, Manassas, VA), was cultured in medium consisting of DMEM, supplemented with 10% FBS, 2% chicken plasma, and 1x P/S.

All chicken cell cultures were maintained at 42°C and 5% CO_2_.

### Primary cell characterization

Chicken PBMSC were characterized based on their (i) spindle-shaped fibroblast-like morphology, (ii) potential for trilineage differentiation, and (iii) immunophenotypical protein profile using flow cytometry, as described previously for equine PBMSC (17, 18). Chicken PBMCs and AECs were used as positive controls to determine cross-reactivity of antibodies against CD45, CD14, and CD34, which are negative markers for MSCs. Semi-quantitative reverse transcription-polymerase chain reaction (RT-PCR) was performed as described before (14) and used to amplify genes encoding the markers CD29, CD172alpha, and CD73, which are positive markers for MSC isolated from other species, but were not detected in chicken PBMSC by the antibodies used in this study.

Chicken AECs were characterized based on (i) their cobblestone morphology, (ii) immunocytochemistry (ICC) staining for the commonly used endothelial cell (EC) marker Von Willebrand factor, and (iii) RT-PCR for EC-associated genes.

Antibodies used for flow cytometry are listed in Table 1 and PCR primer sequences are shown in Table 2.

**Table 1:** Antibodies used for flow cytometry.

**Antibody**	**Clone**	**Dilution**	**Source**	**Cross-reactivity**
CD29	TDM29	1:10–1:50	Chemicon	No
CD90	polyclonal (sheep)	1:50	R&D Systems	Yes
CD172 alpha	DH598	1:10–1:50	WSU/MAC	No
CD44	IM7	1:50	Thermo Fisher	Yes
CD73	polyclonal (sheep)	1:5–1:50	R&D Systems	No
CD73	AA60-E3-3	1:10–1:50	Millipore	No
CD73	H-300	1:5–1:50	Santa Cruz	No
CD105	SN6h	1:50	Thermo Fisher	Yes
CD45	T/29/33	1:10–1:50	Santa Cruz	No
CD45	K252.1E4	1:10–1:50	Thermo Fisher	No
CD14	TUK4	1:50	Bio-Rad	Yes
CD34	QBEND10	1:10–1:50	Thermo Fisher	No
CD34	AV138	1:10–1:50	Bio-Rad	No
CD79a	HM57	1:50	Bio-Rad	Yes

**Table 2:** Primers used for RT-PCR.

CD29	*ITBG1*	AACCAGAGGCCATTACACAG	ATCCACCTTCAGGAGAATCC
CD172 alpha	*SIRPA*	TGAGAGAAGGAAGGAGTGGG	AATGTGCAGTTCAGGGTCAG
CD73	*NT5E*	TTTTGAAGTGAGTCTGGGGC	TTGTGATGGAGCACTGCTAC
endoglin	*ENG*	TCCTGATGCTGAACAACTGC	GTAGGAGGCGATGATGCTGT
cadherin 1	*CDH1*	CCAAAGAAGCCCCTGGACTTCGA	CGTCGGGGTCATGTGCCCAA
selectin E	*SELE*	AATGCAAAGCTGTGACCTGC	GCGTGGATTGTCCTGTCAGA
von Willebrand factor	*VWF*	TCTCGGAGATACAGCCTCAC	CACTTCCTTTTCACCCACAC

### Generation of conditioned medium (CM)

To generate CM, 1 × 10^6^ PBMSC were plated in a T75 flask. After 24 h (h), culture medium was removed, cell monolayers were rinsed twice with PBS, and 8 ml DMEM was added to the flask for CM. CM was collected after 18–20 h, centrifuged twice at 300 × g for 5 min at room temperature (RT) to remove cellular debris, and then applied to target cells. Frozen-thawed (F/T) PBMSC CM was made by freezing fresh CM at −20°C, followed by thawing at RT. Chicken PBMSCs used as a source of CM for antibacterial assays were cultured for at least 3 passages in antibiotic-free medium before being plated for CM generation, to ensure no residual antibiotics were present.

### Antimicrobial assays

*Escherichia (E.) coli* strain 10536, *Staphylococcus* (*S.) aureus strain* 25923, and *methicillin-resistant S. aureus* (*MRSA)* strain USA300 (ATCC, Manassas, VA) were maintained and plated, as previously described (14). CM-bacteria co-culture experiments were conducted, as described previously but with adding 5 × 10^3^ colony forming units (cfu) bacteria to each well and incubating plates for 8 or 24 h at 37°C in an Infinite 200 Pro plate reader (Tecan, Morrisville, NC) (14). Absorbance readings were taken at 600 nm every 30 min. At the end of the 24 h incubation experiments, bacteria were serially diluted and plated on agar plates to quantify CFU/ml in each well.

Biofilm assays were set up with *MRSA*, and absorbance of solubilized crystal violet was detected at 570 nm using an Infinite 200 Pro plate reader (Tecan), as previously described (15).

Each test condition was run in quadruplicate within an assay and all assays were performed 3 times.

### Fibroblast assays

The adhesion strength of chicken fibroblasts to culture wells was quantified using a centrifugation assay that relies on a controlled detachment force, as previously described (29, 30). Absorbance was measured at 570 nm using an Infinite 200 Pro plate reader (Tecan).

To evaluate migration, scratch assays with chicken fibroblasts were set up and carried out, as previously described (16). Photographs of scratches were taken at 0 and 24 h post scratching using an inverted CKX41 light microscope with an Infinity 2 camera (Olympus, Waltham, MA). Migration distances of cells were measured in a blinded manner using ImageJ software (http://imagej.nih.gov/ij). Fibroblast migration was also assessed using Oris^TM^ cell migration assays, according to manufacturer's instructions. Control wells were used to determine the cell-free area at the time of stopper removal. Cells were stained with a crystal violet solution (0.5% crystal violet in 20% [v/v] methanol) for 10 min, washed 2x with distilled water and dried overnight. Images of wells were taken with an inverted CKX41 light microscope with an Infinity 2 camera (Olympus) and cells that migrated into the cell-free area during the assay were counted by a blinded observer.

Each test condition was run in triplicate within an assay and all assays were performed 3 times.

### Angiogenesis assay

Abcam angiogenesis assays were carried out according to manufacturer's instructions. Briefly, chicken AECs were plated on extracellular matrix-coated wells of a 96-well plate and then incubated with either control media, PBMSC CM, fibroblast CM (non-stem cell control), or suramin (inhibitor of angiogenesis) for 18 h. Cells were carefully washed, labeled with staining dye, and imaged using a Zoe fluorescent imager at 20x magnification (Bio-Rad, Hercules, CA). Three images from each well were analyzed using the Angiogenesis Analyzer plug in for ImageJ software (31). Each test condition was run in triplicate wells within an assay and all assays were performed 3 times.

### Statistical analysis

One-way ANOVA, followed by the Tukey's multiple comparison test was used to determine statistically significant differences in CFU/ml bacteria and absorbance values in bacterial assays, migration and adhesion in fibroblast assays, and number of branches and mesh index in angiogenesis assays (*P* < 0.05). In assays where absorbance values or number of migrated cells were expressed as the percentage of control, values from the technical replicates of the control wells on the one hand and the treatment wells on the other hand were averaged and following equation was used to determine percentage of control: (treatment avg^*^100)/control avg = % control. GraphPad software was used for analysis. Data given are the mean of 3 replicates and bars show standard deviations.

## Results

### Isolation and characterization of chicken peripheral blood-derived mesenchymal stromal cells (PBMSCs) and aortic endothelial cells (AECs)

**Figure 1:**
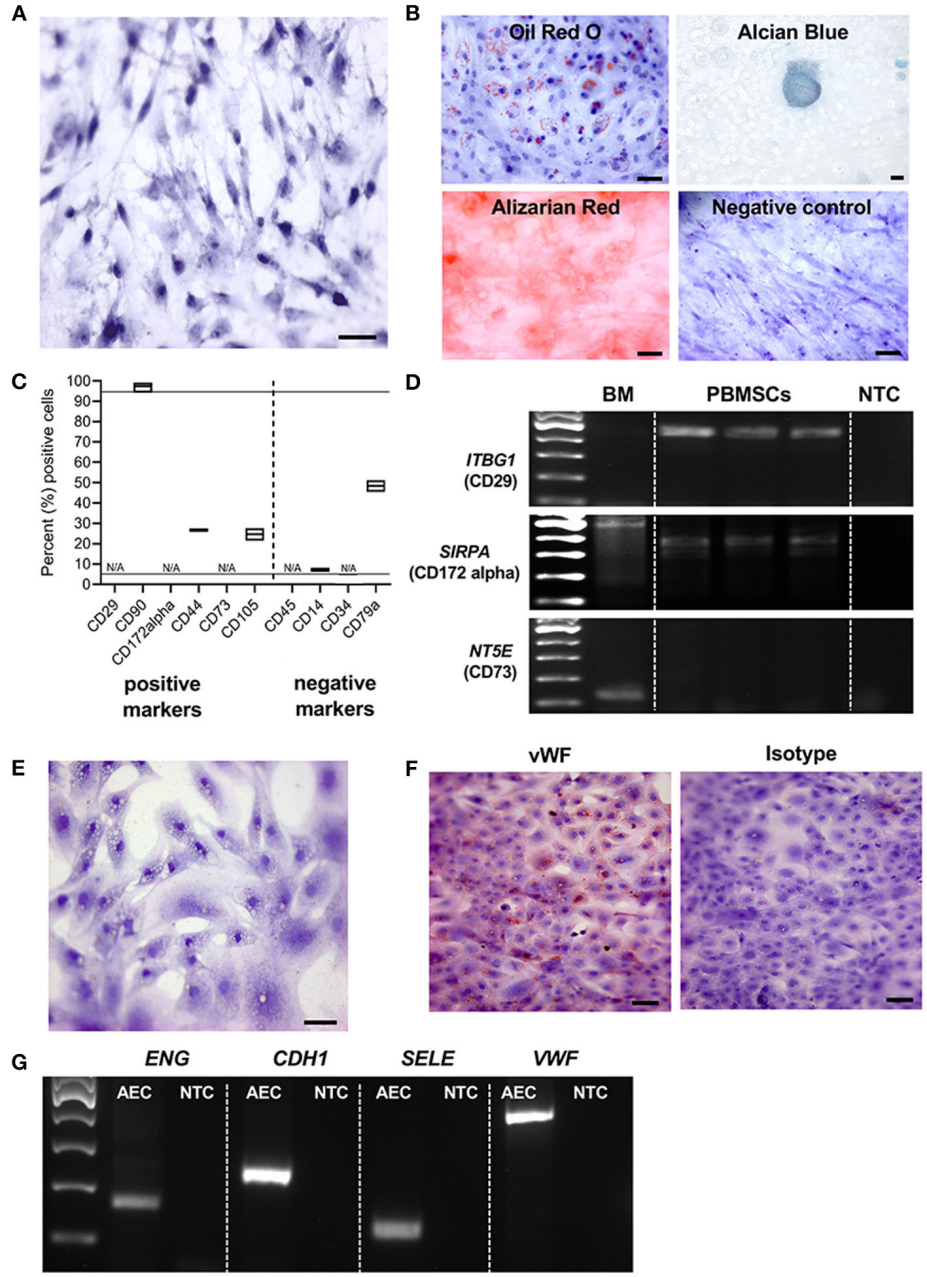
Characterization of chicken peripheral blood-derived mesenchymal stromal cells (PBMSCs) and aortic endothelial cells (AECs). **(A)** Brightfield image of chicken PBMSCs counterstained with hematoxylin. **(B)** Images of chicken PBMSCs after *in vitro* differentiation into adipocytes (oil red O), chondrocytes (alcian blue), and osteocytes (alizarin red). Undifferentiated cells are shown as controls (hematoxylin). **(C)** Cellular expression patterns of proteins determined by the International Society for Cellular Therapy (ISCT) to be used for MSC immunophenotyping, as detected by flow cytometry of chicken PBMSC. N/A indicates antibody specificity is undetermined *n* = 3. **(D)** Amplification of the genes *ITBG1* (encoding CD29), *SIRPA* (encoding CD172 alpha), and *NT5E* (encoding CD73) from chicken PB-MSCs. Chicken bone marrow (BM) was used as a positive control. NTC = no template control *n* = 3. **(E)** Brightfield image of chicken AECs counterstained with hematoxylin. **(F)** Images of chicken AECs labeled with an antibody recognizing von Willebrand factor (vWF) and an isotype control. **(G)** Amplification of the genes *ENG, CDH1, SELE* and *VWF* in chicken AECs. NTC, no template control. Scale bars = 50 μm.

We are the first to report the successful isolation and characterization of MSCs derived from chicken whole blood. Chicken PBMSCs were plastic adherent and exhibited the typical spindle-shaped fibroblast-like MSC morphology in culture (Figure 1A). Like MSCs from other species, chicken PMBSCs differentiated into adipocytes, chondrocytes, and osteocytes, when cultured in appropriate differentiation media (Figure 1B).

Immunophenotyping of chicken PBMSCs by flow cytometry showed that the cells were positive for some canonical markers of human MSCs, as determined by the International Society of Cellular Therapy (32), and negative for CD14, a monocyte marker (Figure 1C). As there are few descriptions of commercially available antibodies that cross-react with chicken proteins, we chose antibodies based on prior reports (3, 12) and/or antigen homology. When available, we evaluated multiple clones/variations of the antibodies (Table 1), but we were unsuccessful in finding cross-reacting antibodies for the MSC markers CD29, CD172alpha, and CD73 (“N/A,” Figure 1C). Using RT-PCR, we detected expression of the genes *ITBG1* (encoding CD29) and *SIRPA* (encoding CD172 alpha), but not *NT5E* (encoding CD73) in chicken PBMSCs (Figure 1D). *NT5E* was amplified from chicken bone marrow cDNA, indicating that the PCR primers for this gene were functional (Figure 1D). MSCs should not express CD45, CD14, and CD34, and we did not detect these proteins in chicken PBMSCs by flow cytometry (Figure 1C). To determine if this lack of detection was due to the true absence of protein or rather the antibodies not cross-reacting with chicken, we performed flow cytometry using positive control cells, including chicken peripheral blood mononuclear cells (PBMCs) for CD45 and CD14, and chicken aortic endothelial cells (AECs, characterization described in the next paragraph) for CD34. Expression of CD45 and CD34 were not detected in the respective positive control cells, indicating that the antibodies did not cross-react with chicken proteins, whereas CD14 expression was detected in chicken PBMCs (Supplemental Figure 1), confirming that chicken PBMSCs are truly negative for this marker (Figure 1C). Expression of CD79a in chicken PBMSCs was detected by flow cytometry, despite this marker being reported to be absent in human MSCs (32) (Figure 1C), indicating species-specific differences in MSC marker expression, as has been reported previously by us and others for equine and canine MSCs (33, 34).

AECs isolated from fetal chicken aortic blood vessels exhibited the cobblestone morphology typical of endothelial cells (Figure 1E). They were further characterized by protein expression of von Willebrand factor (vWF) using immunocytochemistry (ICC) (Figure 1F) and gene expression of *ENG* (encoding endoglin), *CDH1* (encoding cadherin 1), *SELE* (encoding selectin E) and *VWF* (encoding von Willebrand factor), using RT-PCR (Figure 1G), all of which are characteristically expressed by endothelial cells.

Chicken peripheral blood-derived mesenchymal stromal cell (PBMSC) conditioned medium (CM) selectively inhibits planktonic bacterial growth.

**Figure 2:**
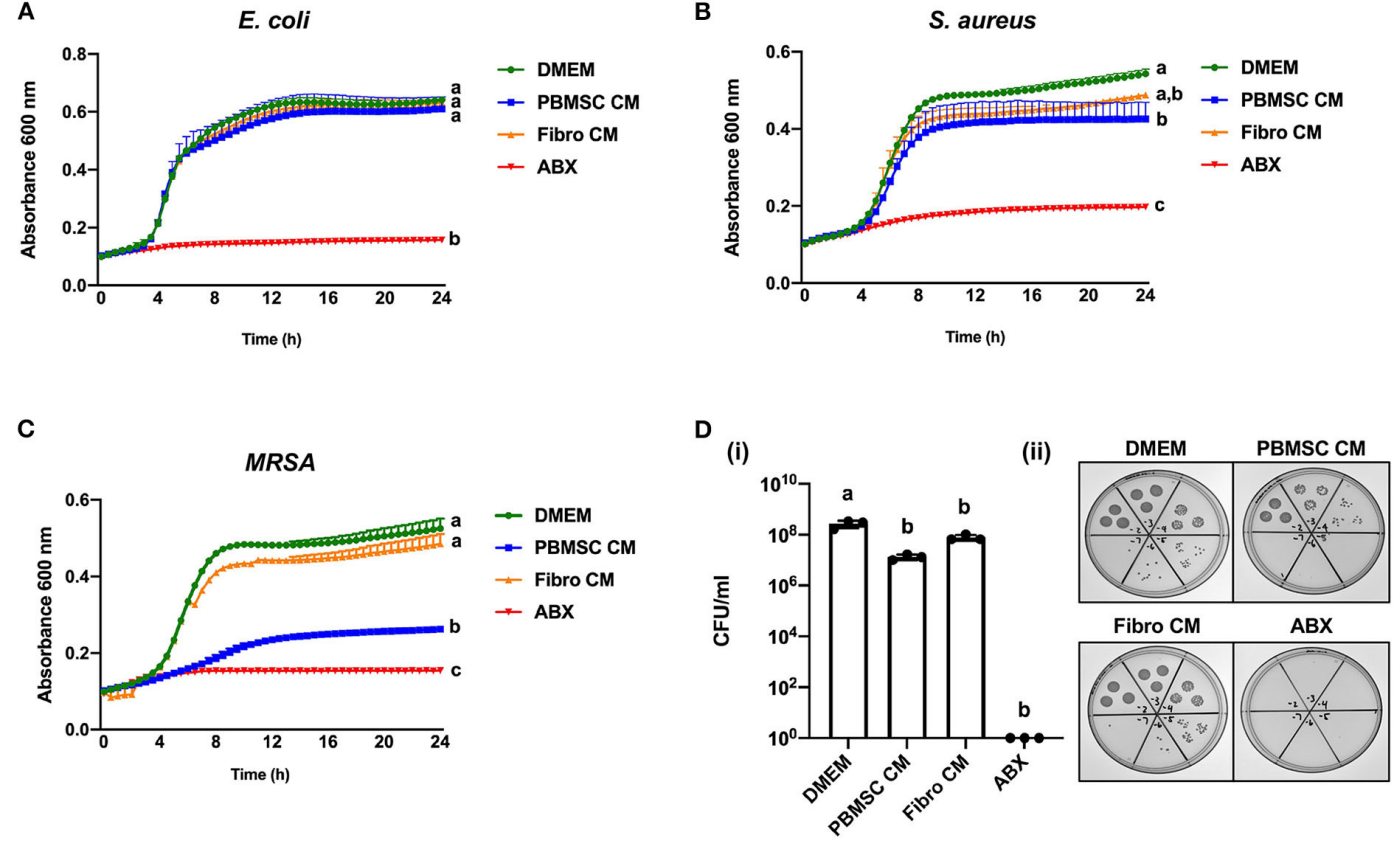
Conditioned medium (CM) collected from chicken peripheral blood-derived mesenchymal stromal cells (PBMSCs) selectively inhibits planktonic bacterial growth. Growth curves of *E. coli* **(A)**, *S. aureus* **(B)**, and *MRSA* **(C)** cultured in DMEM (control medium), PBMSC CM, fibroblast (Fibro) CM, and antibiotics (ABX) for 24 h. **(D)** Colony forming units (CFU) per ml *MRSA* collected after 8 h of culture in DMEM, PBMSC CM, Fibro CM, and ABX. (i) Representative images of *MRSA* colonies. (ii) *n* = 3. Different letters indicate statistically significant differences (*P* < 0.05).

We have shown previously that equine PBMSCs secrete bioactive factors that inhibit the growth of bacteria in planktonic cultures, suggesting that the MSC secretome may be used as an alternative to conventional antibiotics in veterinary medicine (14, 15). When performing similar experiments with CM collected from chicken PBMSC cultures, we found no effect of chicken PBMSC CM on the growth of *E. coli*, but a small, yet significant effect on *S. aureus* growth after 24 h (Figures 2A,B). An even larger significant inhibitory effect on the growth of planktonic *MRSA* was observed after 24 h in the presence of chicken PBMSC CM, when compared to the negative controls consisting of DMEM and CM collected from chicken fibroblasts (Figure 2C). The inhibitory effect of PBMSC CM as compared to DMEM was supported by significantly reduced colony forming units (CFU)/ml of *MRSA* at the end of the assays (Figure 2D).

### Chicken peripheral blood-derived mesenchymal stromal cell (PBMSC) conditioned medium (CM) inhibits planktonic MRSA growth, but does not affect biofilm formation

**Figure 3:**
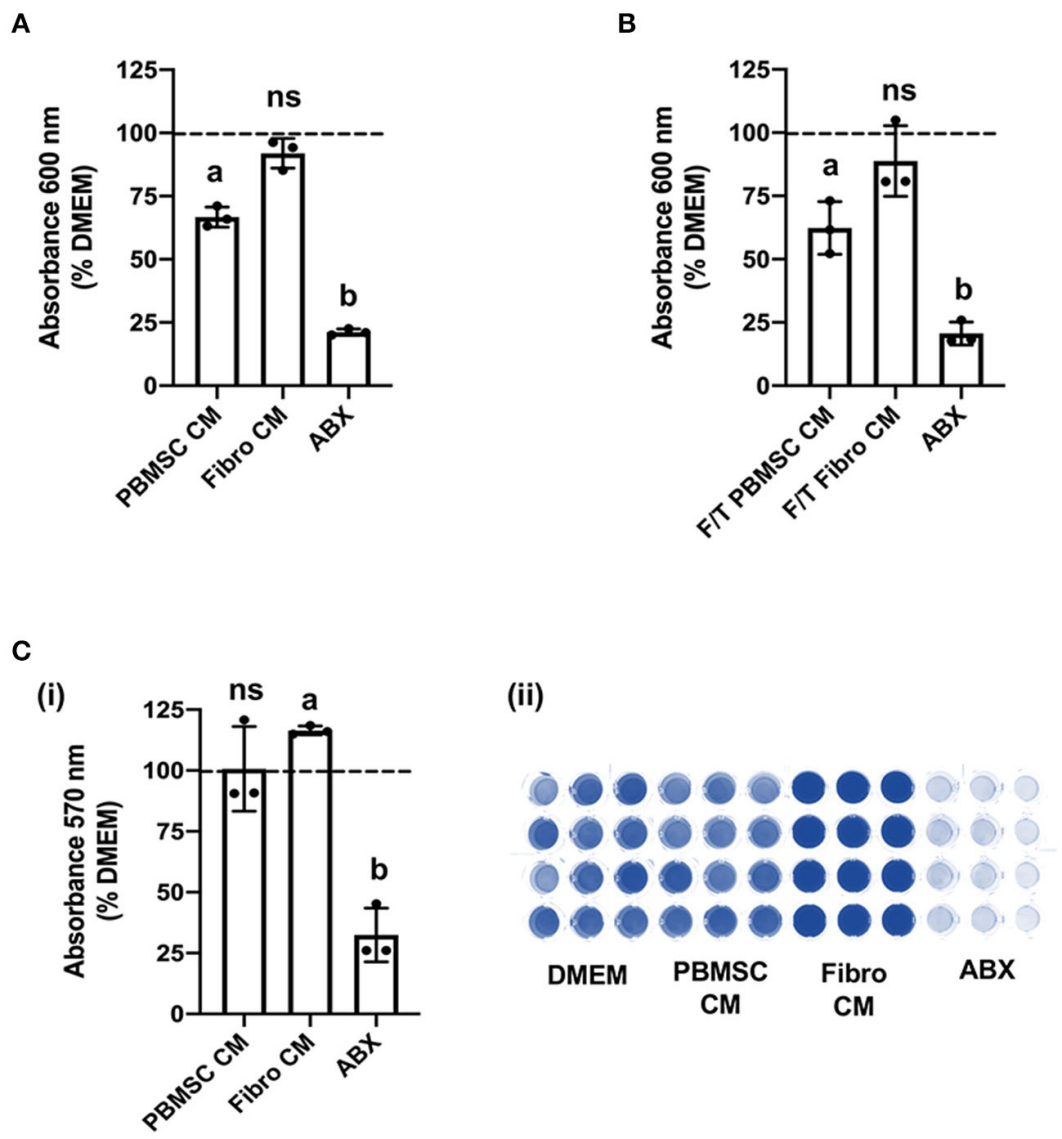
Conditioned medium (CM) collected from chicken peripheral blood derived mesenchymal stromal cells (PBMSCs) inhibits planktonic *MRSA* growth, but does not affect biofilms. **(A)** Growth of *MRSA* after 8 h cultured in DMEM (control medium), PBMSC CM, fibroblast (Fibro) CM, and antibiotics (ABX). **(B)** Growth of *MRSA* after 8 h cultured in DMEM, frozen-thawed (F/T) PBMSC CM, F/T Fibro CM, and ABX. **(C)** Growth of *MRSA* biofilms after 48 h cultured in DMEM, PBMSC CM, Fibro CM, and ABX. (i) Representative images of biofilms stained with crystal violet and solubilized. (ii) *n* = 3. Different letters indicate statistically significant differences (*P* < 0.05).

Based on the encouraging results with planktonic *MRSA*, we decided to explore the antibacterial effect of chicken PBMSC CM on this pathogen in more depth using 8-h antimicrobial assays. First, we confirmed that chicken PBMSC CM significantly inhibited the growth of planktonic *MRSA* at that time point (Figure 3A). We then repeated these experiments with frozen-thawed (F/T) chicken PBMSC CM and found a similar effect on *MRSA*, suggesting that the factor(s) responsible for inhibiting bacterial growth are freeze/thaw resistant (Figure 3B). In contrast, chicken PBMSC CM did not significantly inhibit the formation of *MRSA* biofilms (Figure 3C), indicating that while chicken PBMSC CM effectively reduces *MRSA* growth under planktonic conditions, it does not interfere with biofilm formation.

### Chicken peripheral blood derived mesenchymal stromal cell (PBMSC) conditioned medium (CM) decreases adhesion and promotes migration of fibroblasts

**Figure 4:**
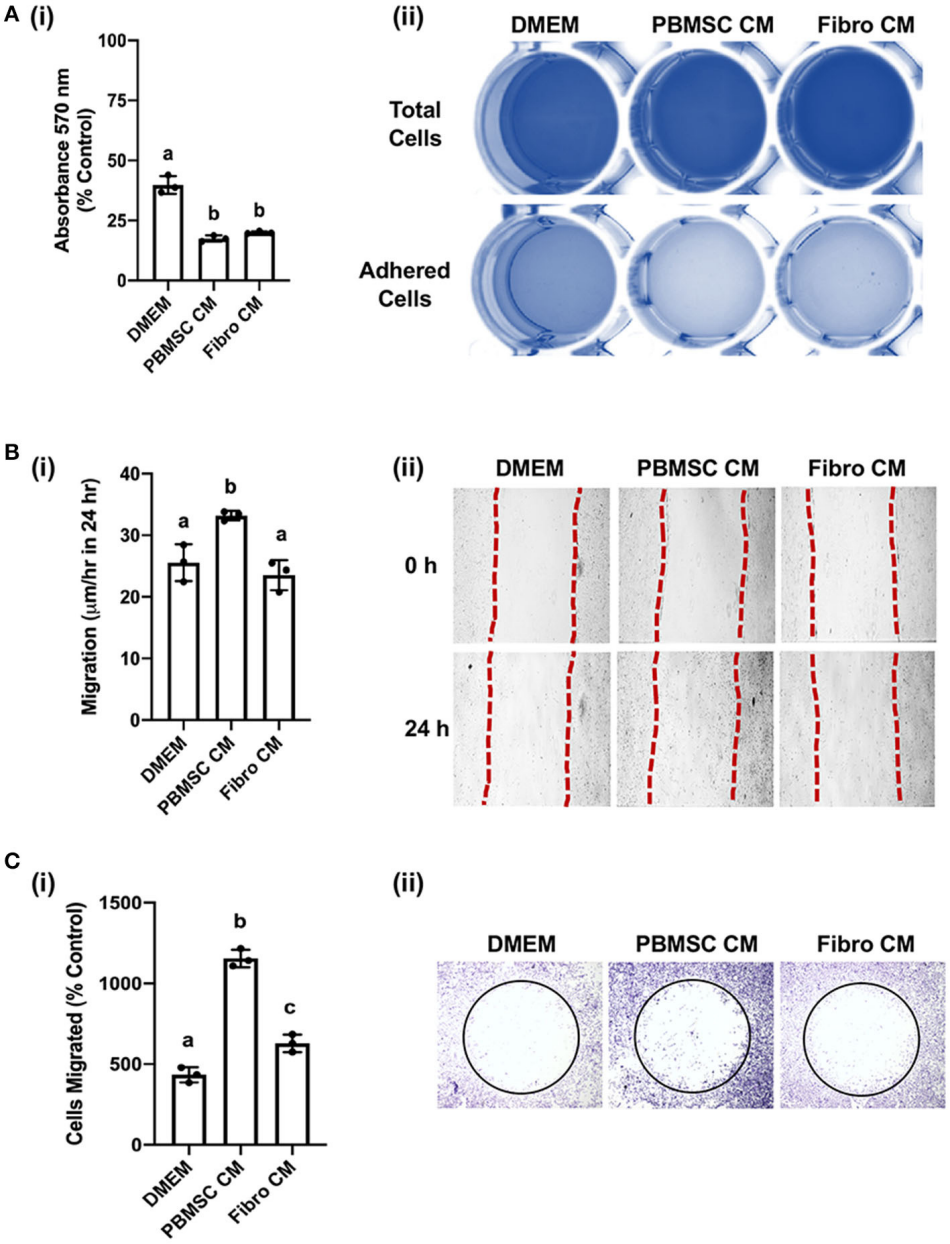
Conditioned medium (CM) collected from chicken peripheral blood derived mesenchymal stromal cells (PBMSCs) decreases fibroblast adhesion and promotes fibroblast migration. **(A)** Adhesion of chicken fibroblasts after culture in DMEM (control medium), PBMSC CM, and fibroblast (Fibro) CM. (i) Representative images of chicken fibroblasts stained with crystal violet and solubilized at the end of the adhesion assay. (ii) **(B)** Migration of chicken fibroblasts cultured in DMEM, PBMSC CM, and Fibro CM in an *in vitro* scratch assay. (i) Representative images of chicken fibroblasts in an *in vitro* scratch assay taken at the time of scratching (0 h) and 24 h post scratching. **(C)** Numbers of chicken fibroblasts migrated in an Oris^TM^ cell migration assay. (i) Images of crystal violet stained chicken fibroblasts in an Oris^TM^ cell migration assay. (ii) *n* = 3. Different letters indicate statistically significant differences (*P* < 0.05).

We previously demonstrated that equine PBMSC CM acts on fibroblasts by decreasing adhesion strength and increasing migration potential, two related cell characteristics that contribute to wound healing (16, 17). Similar effects were observed with chicken PBMSC CM in the present study. Specifically, chicken fibroblasts adhered less strongly to culture wells in the presence of chicken PBMSC CM when compared to DMEM, as visualized by a lower uptake of crystal violet per well after centrifugation, but adhesion was similar to that of chicken fibroblasts cultured in the presence of chicken fibroblast CM (Figure 4A). Since decreased adhesion is correlated with increased cell migration in culture, we used an *in vitro* scratch assay and found that chicken fibroblasts cultured in the presence of chicken PBMSC CM migrated a greater distance in 24 h than fibroblasts cultured in DMEM or fibroblast CM (Figure 4B). This result was corroborated using an Oris^TM^ cell migration assay (Figure 4C), which relies more on the number of cells that move, rather than the distance moved, to determine cell migration.

### Chicken peripheral blood derived mesenchymal stromal cell (PBMSC) conditioned medium (CM) increases the number of branches and mesh index of aortic endothelial cells (AECs)

**Figure 5:**
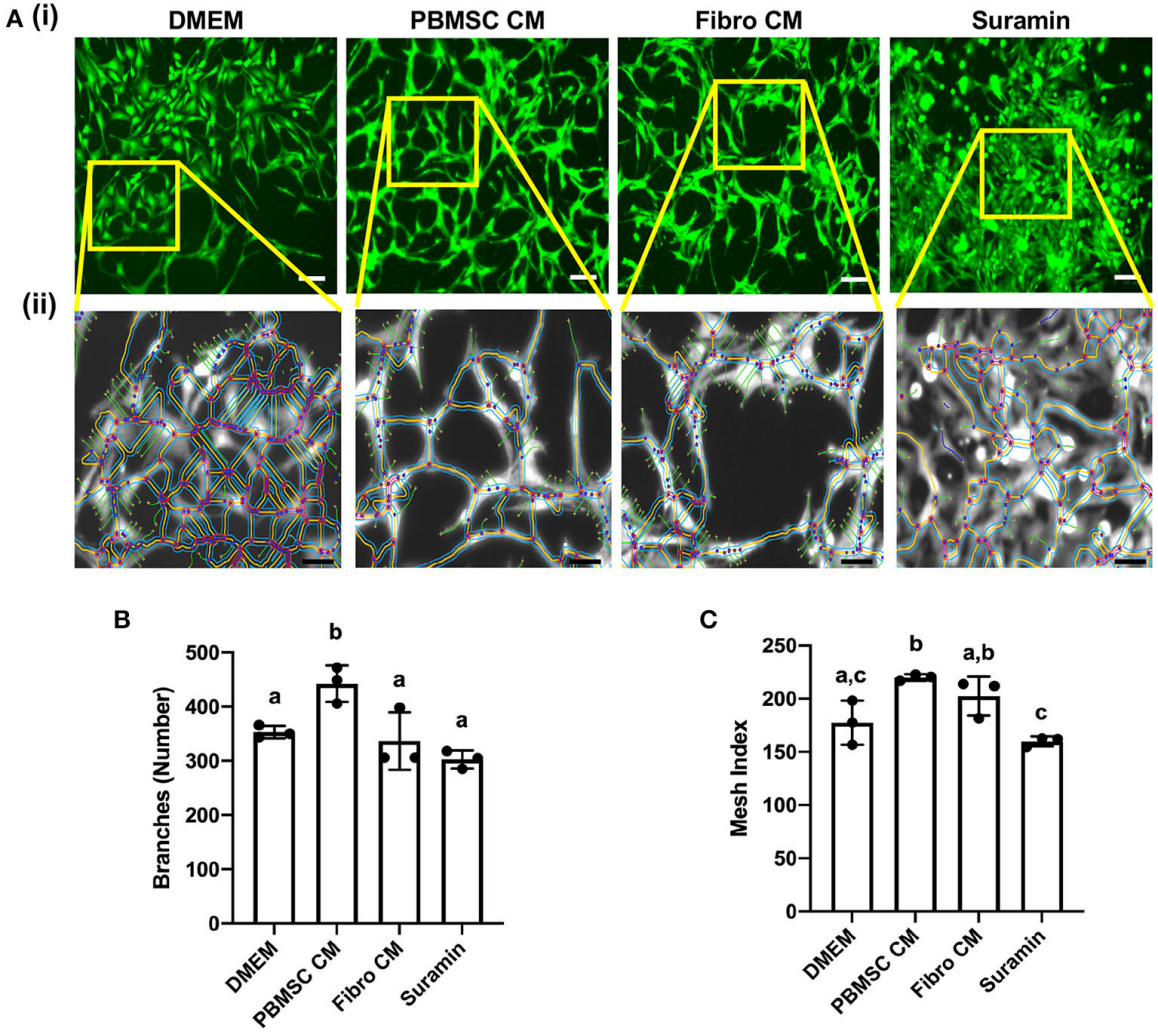
Conditioned medium (CM) collected from chicken peripheral blood derived mesenchymal stromal cells (PBMSCs) increases the number of branches and mesh index of aortic endothelial cells (AECs). **(A)** Images of stained chicken AECs in angiogenesis assays. (i) Expanded view of boxed regions, showing measurements made by the Angiogenesis Analyzer, used to determine numbers of branches, mesh index and additional features of angiogenesis. (ii) Numbers of branches **(B)** and mesh index **(C)** of AECs cultured in DMEM (control medium), PBMSC CM, fibroblast (Fibro) CM, and suramin (negative control) for 18 h. *n* = 3. Scale bars = 50 μm. Different letters indicate statistically significant differences (*P* < 0.05).

An additional functional effect of equine PBMSC CM we reported previously, is the impact MSC secreted factors have on endothelial cells (18). Here, we performed an angiogenesis assay with chicken AECs in the presence of chicken PBMSC CM and analyzed various aspects of angiogenesis using the Angiogenesis Analyzer tool plug in for ImageJ software (Figure 5A). We found that PBMSC CM increased the number of branches formed by AECs, as well as the mesh index, when compared to AECs cultured in the presence of DMEM and fibroblast CM, which behaved more like AECs cultured with the negative control suramin (Figures 5B,C). Other indicators of angiogenesis were not significantly different when AECs were cultured in PBMSC CM as compared to the control conditions (data not shown).

## Discussion

The potential of mammalian mesenchymal stromal cells (MSCs) to improve wound skin wound healing and inhibit bacterial growth has been well-studied *in vitro* (35, 36). Encouraging results have led to small-scale pilot studies and larger experiments *in vivo*, designed to test the efficacy of MSCs as a treatment for skin wounds, including reducing the bacterial load in wounds, in mammals (37–40). Far less is known about MSCs isolated from birds. Only a few groups have reported on the isolation of MSCs from turkeys and ducks (41–43), and although more research has been done studying the characteristics of MSCs derived from chickens (44), the effects of chicken MSCs, in particular the chicken MSC secretome, on wound healing has not been explored.

This study is the first to isolate and characterize chicken MSCs from the peripheral blood and the first to describe the effects of the chicken PBMSC secretome, collected as CM, on the growth of various bacteria and target cell types found in the skin. Our novel findings are that the chicken PBMSC secretome (i) inhibits the growth of planktonic *Staphylococcus aureus* (*S. aureus*), and even more significantly the methicillin resistant *S. aureus* (*MRSA*), (ii) decreases adhesion and promote migration of fibroblasts, and (iii) supports endothelial cell tube formation. Exploring the potential of chicken PBMSC CM as a novel biological for the treatment of cutaneous diseases of poultry has direct relevance to gangrenous dermatitis (GD) and pododermatitis or bumblefoot (19, 24), where currently available preventatives and treatments could benefit from an adjunct or combination therapy with PBMSC secretome, that could be delivered topically or by intradermal injection, based on previously published data on the use of MSCs and/or MSC CM to treat skin wounds (37, 39).

The therapeutic use of chicken PBMSC CM to inhibit the growth of *S. aureus*, and in particular *MRSA*, could benefit both chicken and human health. *S. aureus* is a bacterial pathogen of humans and other animals, and methicillin-resistant strains cause severe diseases in humans. *MRSA* has been isolated from live broiler chickens (45), bioaerosols from a chicken farm (46), and chicken meat procured from retail stores (47). Moreover, *MRSA* can be transmitted from chickens to humans by direct contact with live animals, as spillover from nearby farms (48), and through food products (49), and *MRSA* has been reported to be a causative agent in avian GD and pododermatitis (50, 51). Reducing *S. aureus* and *MRSA* on poultry farms, by directly inhibiting bacterial growth without use of conventional antibiotics, could, therefore, help prevent spread of these pathogens without applying pressure that could promote additional antibiotic resistance.

In our previous work with equine PBMSCs, we found that secreted factors inhibited the planktonic growth of S. *aureus, E. coli, P. aeruginosa, A. viridans, A. baumannii*, and *MRSA*. In addition, the equine PBMSC secretome reduced biofilms formed by S. *aureus, E. coli, A viridans, A. baumannii*, and *MRSA* (14, 15). For the current study, we looked at the effects of chicken PBMSC CM on limited species of bacteria, and found that it inhibits the planktonic growth of *S. aureus* and *MRSA*, but did not inhibit the growth of planktonic *E. coli* nor had an effect on *MRSA* biofilm formation. Future experiments will explore the effects of PBMSC CM on the growth of additional bacteria species associated with GD and pododermatitis, including select species from the genera *Clostridium* and *Staphylococcus*. We also plan to conduct experiments to identify the bioactive factors in chicken PBMSC CM that inhibit bacterial growth, such as -omics screens. We have previously used antibody-based methods to show that equine PBMSC secrete (i) the antimicrobial peptides cystatin C, elafin, lipocalin 2, and cathelicidin that inhibit the growth of planktonic bacteria, and (ii) cysteine proteases that degrade proteins in *MRSA* biofilms, making them more susceptible to antibiotic treatment (14, 15). Determining the underlying mechanisms mediating the antimicrobial activity of chicken PBMSCs will help us understand what bacterial species are likely to be targeted by chicken PBMSC secreted factors and how these factors can be applied most effectively as a therapy.

The effects of the chicken PBMSC secretome on target cells in the skin were largely similar to those we previously reported with equine PBMSC (16–18), and consisted of reducing fibroblast adhesion to substrates, increasing fibroblast migration, and promoting angiogenesis *in vitro*. Although wound healing models in chicken corneas and chicken embryos are well established (52, 53), there is currently a lack of literature focusing on cutaneous wound models in adult chickens. Developing such a model and using it to determine the effects of chicken PBMSC secreted factors on skin wound healing would expand our understanding of the mode-of-action of chicken PBMSC secreted factors, and help us to determine the best ways to deliver them to wounded chickens.

Collectively, the *in vitro* experiments in this study demonstrate that chicken PBMSCs secrete bioactive molecules that inhibit the growth of bacteria, stimulate skin fibroblasts and act on endothelial cells. These data provide a solid basis for future laboratory experiments designed to identify the factors secreted by chicken PBMSCs that could be used to improve wound healing, as wells as *in vivo* trials to test the efficacy of chicken PBMSC CM as a therapy for cutaneous disease of chickens.

## Data availability statement

The original contributions presented in the study are included in the article/Supplementary material, further inquiries can be directed to the corresponding authors.

## Ethics statement

The animal study was reviewed and approved by Cornell Institutional Animal Care and Use Committee (IACUC # 2014-0038).

## Author contributions

RH and GV conceived and designed the study and wrote the manuscript. RH, KC, and SP performed the experiments. RH, KC, and GV interpreted the data. All authors reviewed this manuscript and approved the final version.

## Funding

This study was funded by the National Institute of Food and Agriculture, U.S. Department of Agriculture through a Hatch Grant # NYC-473426.

## Conflict of interest

The authors declare that the research was conducted in the absence of any commercial or financial relationships that could be construed as a potential conflict of interest.

## Publisher's note

All claims expressed in this article are solely those of the authors and do not necessarily represent those of their affiliated organizations, or those of the publisher, the editors and the reviewers. Any product that may be evaluated in this article, or claim that may be made by its manufacturer, is not guaranteed or endorsed by the publisher.

## Supplementary material

Supplementary Figure 1 Flow cytometry controls for CD45, CD14 and CD34. Flow cytometry plots of chicken peripheral blood mononuclear cells (PBMCs) labeled with CD45 and CD14 antibodies and chicken aortic endothelial cells (AECs) labeled with CD34 antibody.
