# Supplementary figures and images for: Mesenchymal stromal cells isolated from chicken peripheral blood secrete bioactive factors with antimicrobial and regenerative properties
Source: Front Vet Sci. 2022 Aug 24;9:949836. doi: 10.3389/fvets.2022.949836 (PMC9449329; doi:10.3389/fvets.2022.949836)

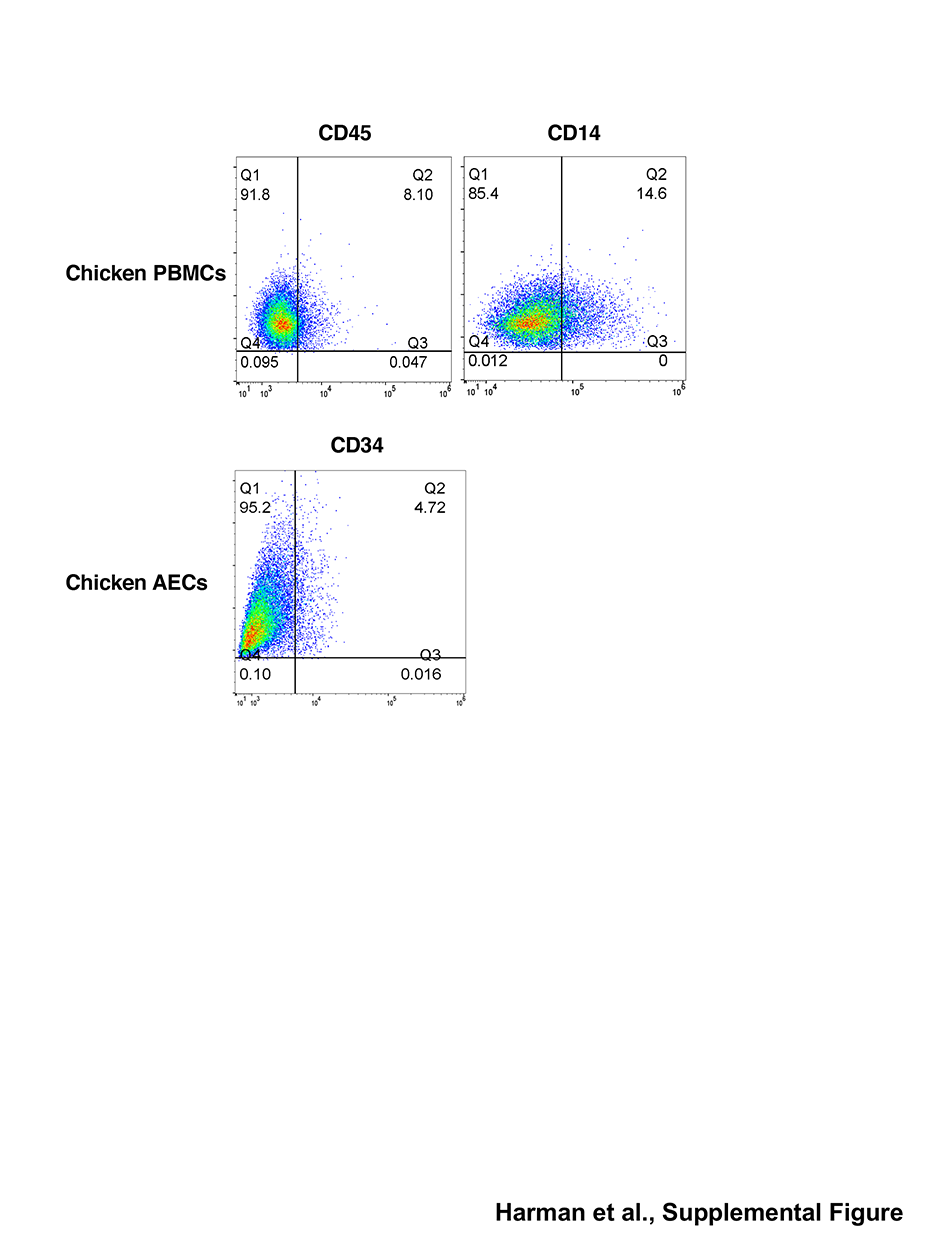

Supplement: Supplementary Figure 1 — Flow cytometry controls for CD45, CD14 and CD34. Flow cytometry plots of chicken peripheral blood mononuclear cells (PBMCs) labeled with CD45 and CD14 antibodies and chicken aortic endothelial cells (AECs) labeled with CD34 antibody. [file Image_1.TIF]
